# Animal Castration

**Published:** 1884-10

**Authors:** 


### REVIEWS.

ANIMAL CASTRATION. By A. Liautard, M.D., etc. New York: W. R. Jenkins, 850 6th Avenue, pp. 148.

It is a great pleasure indeed to review a book of this kind. All engaged in its production are fully competent and do themselves justice. The book is beautifully printed, with large type, on good paper, and is profusely illustrated with excellent wood cuts. Dr. Holt is credited with revising the manuscript, and either he or the author, or both, must be masters of the English language. It is rare to find the text of a book so clear and wonderfully well expressed. The author is evidently master of his subject, and the castration of all domestic animals from horses and cattle down to dogs, pigs, and sheep, both males and females, and the caponing of cocks and hens is detailed in a way that leaves nothing to criticise and everything to praise.

We can not say less, we need not say more except of course that every one interested in the subject of castration should buy the book. Another edition will doubtless soon be called for, but we doubt whether any improvements can be made.

